# Effect of internal and external cooling on high‐intensity intermittent cycling performance and cognitive function in the heat

**DOI:** 10.1113/EP092679

**Published:** 2025-09-27

**Authors:** Stacey Cowe, Simon B. Cooper, Rachel Malcolm, Caroline Sunderland

**Affiliations:** ^1^ Department of Sport Science, Sport, Health and Performance Enhancement (SHAPE) Research Centre Nottingham Trent University Nottingham UK

**Keywords:** cognitive function, cooling, sprint performance

## Abstract

We investigated the effect of internal and external cooling on high‐intensity intermittent cycling performance and cognitive function in the heat. Twenty‐nine males completed a control trial (CON) and a cooling trial (ice slurry and ice collar; COOL) in the heat (33°C, 50% relative humidity) involving a 40 min intermittent cycling protocol (two sets of ten 2 min stages, each consisting of 5 s sprint, 105 s active recovery and 10 s rest). A battery of cognitive tests was completed pre‐ and postexercise, with physiological and perceptual responses recorded throughout. No differences in peak or mean power output were found between conditions (all *p *> 0.05). Average trial rectal (COOL: 37.39°C ± 0.59°C; CON: 37.59°C ± 0.56°C, *p *< 0.001) and neck (COOL: 28.87°C ± 4.87°C; CON: 32.82°C ± 1.43°C, *P *< 0.001) temperatures were found to be lower in COOL. Participants reported feeling better and reported lower ratings of thermal sensation and improved comfort in COOL (all *p *< 0.05). Response times on the Stroop task complex level were quicker over time in COOL (COOL: −48 ± 23 ms; CON: −11 ± 18 ms, *p *= 0.002) and quicker overall on the number level of Sternberg during COOL (COOL: 434 ± 77 ms; CON: 437 ± 84 ms, *p* = 0.046). However, over time, the improvement in response times on the number level of Sternberg was greater in CON (COOL: −6 ± 3 ms; CON: −26 ± 2 ms, *p *= 0.015). Response times became quicker over time to a greater extent in CON on the visual search complex level (COOL: −15 ± 1 ms; CON: −119 ± 31 ms, *p *= 0.009). The combined cooling intervention did not influence sprint performance and had only a minimal influence on some domains of cognitive function but did lead to improvements in physiological and perceptual responses. These findings provide information on a practical combined cooling method that can be implemented in elite sport.

## Abstract (highlights)

- **What is the central question of this study?**
  The ability to repeatedly perform high‐intensity efforts interspersed with minimal recovery, coupled with optimal cognitive function, is imperative for successful team‐sport performance. However, both components face impairment when core temperatures reach 38.5°C, which can occur commonly when exercising in hot environments.
- **What is the main finding and its importance?**
  The combined cooling intervention was successful at lowering rectal and neck temperature and reducing heart rate. Additionally, the combined cooling intervention elicited lower ratings of thermal sensation, improved comfort and lower perceived exertion. Despite this, no differences were found in sprint performance.

## 1 INTRODUCTION

The ability to perform repeated sprints and high‐intensity, intermittent efforts interspersed with minimal recovery is one of the most crucial physical components of team sport performance (Spencer et al., 2005). In sports such as football and rugby, athletes are required to cover distances of ≤10 km in a single match, combined with frequent, intermittent bursts of activity that include sprinting and jumping (Meir et al., 1993; 2001; Stølen et al., 2005). However, repeated‐sprint ability has been found to be impaired when core temperatures are ≥38.5°C (Drust et al., 2005; Girard et al., 2015; Sunderland & Nevill, 2005). This is an area of concern for team sport athletes, because many major competitions are increasingly being held in hot and humid environments, which causes increases in core temperature and cardiovascular strain and can impair performance (Mazalan et al., 2022a; Périard et al., 2021).

The effects of heat on intermittent sprint performance were evidenced by Drust et al. (2005), who found that peak and mean power output declined to a larger extent across maximal sprints during hot conditions [40.3°C, 17% relative humidity (RH)] compared with control conditions (20°C, 24% RH). A 40 min intermittent cycling protocol was used, consisting of 15 s of loaded cycling and 15 s of rest followed by five 15 s maximal sprints interspersed with 15 s of recovery. This finding was coupled with elevated core and muscle temperatures, which were suggested to be the main mechanisms causing impaired sprint performance. Conversely, no differences in peak power output were found between hot (40°C, 40% RH) and control (24°C, 24% RH) conditions when participants completed eight 6 s maximal cycling sprints, which were separated by 1 min of passive recovery followed by 4 min of constant‐intensity cycling (Almudekhi et al., 2012). However, participants reached a modest level of hyperthermia, because final core temperatures reached an average of 38.2°C ± 0.3°C and were similar across both conditions. Differences in the findings between the two studies might be attributable to the longer duration of intermittent exercise, particularly the maximal sprint element, in the study conducted by Drust et al. (2005), hence resulting in greater physiological strain. However, the inconsistent findings prompt the need for more research whilst also using an ecologically valid protocol that investigates other components of team sport.

Successful team sport performance is reliant on multiple factors in addition to physical components, including a range of cognitive skills and functions (Williams, 2000) that are vital for information processing and decision making. Within football, where time restrictions apply, players must be able to identify vacant space and team‐mate positioning by using their vigilance, selective attention and working memory to play or pass the ball effectively (Heppe et al., 2016). However, this ability to process information might be limited owing to cognitive task performance being susceptible to impairment when the core temperature of an individual reaches or exceeds 38.5°C (Bandelow et al., 2010; Hocking et al., 2001; Kenefick et al., 2007; Schmit et al., 2017). This impairment has been attributed to a reduction in cerebral blood flow, probably attributable to the redirection of blood to the periphery (for cooling) and to the working muscles, which, in turn, might negatively impact heat removal from the brain (Falkowska et al., 2015; Nybo & Nielsen, 2001). Additionally, previous studies have found that negative subjective feelings in the heat, such as thermal sensation, are another potential driving force in the decline of cognitive function (Gaoua et al., 2012). Given the above‐mentioned impairments to both intermittent exercise and cognitive performance, strategies to alleviate these negative effects are required.

When exercising in the heat, single cooling strategies have previously been used to reduce thermal strain, positively influence perceptual responses and enhance exercise performance by delaying thermally induced fatigue (Bongers et al., 2017; Wegmann et al., 2012). Positive effects on cognitive function have also been shown, particularly in tasks of higher complexity including working memory and executive function (Saldaris et al., 2019; Siegel et al., 2010; Tyler & Sunderland, 2011). However, more recently, researchers have highlighted that combined internal and external cooling might have synergistic beneficial effects on lowering core temperature and improving perceptual responses by simultaneously targeting vital regions of the body for cooling (Bongers et al., 2015). Owing to these potential advantages of combined cooling interventions, it is important that combined cooling strategies are researched.

Following an intermittent treadmill running protocol, which simulated tennis match play, in the heat (36.5°C, 50% RH), a combined cooling strategy consisting of a cooling vest, neck collar and cold sports drink was successful at increasing the time to cessation of exercise and in improving executive function (Wen et al., 2022). These findings were coupled with lower gastrointestinal temperature alongside reduced thermal sensation and rate of perceived exertion (Wen et al., 2022). Previous research (Duffield et al., 2009; Minett et al., 2012) has also found improved exercise performance in the heat, which was evidenced through increased running distances, following pre‐cooling with combined cooling interventions. These interventions consisted of head/neck cooling, an ice vest and hand immersion (Minett et al., 2012) or cooling vests, cold neck towels and ice packs on the quadriceps (Duffield et al., 2009). Despite these positive findings, more research is required that investigates the effect of a combined cooling intervention on a wider range of cognitive domains that are relevant to team sport whilst also examining the effect on intermittent exercise in the heat.

Therefore, the aim of the present study was to investigate the effect of internal and external cooling on high‐intensity intermittent cycling performance and cognitive function in the heat. It was hypothesized that the use of a combined cooling intervention would reduce the negative impact of the heat on intermittent sprint performance by minimizing physiological strain and improving perceptual responses. Moreover, it was hypothesized that the combined cooling would also attenuate any detriments to cognition during high‐intensity intermittent exercise in the heat.

## 2 MATERIALS AND METHODS

### 2.1 Participants

Twenty nine unacclimatized male games players (age: 21.97 ± 2.57 years; body mass: 79.53 ± 9.91 kg; height: 180.97 ± 5.76 cm; maximal oxygen uptake: 44.58 ± 5.90 mL/kg/min) volunteered to participate in the present study after providing written informed consent and completing a health screen questionnaire. Written and verbal information regarding the study was given to the participants, in addition to an opportunity to ask questions. Only males were recruited for this study owing to time constraints of the winter period and Christmas vacation and the time necessary to test around the menstrual cycle not being available. The human invasive ethical advisory committee of Nottingham Trent University granted approval for the present study (reference number: 1614212), and the standards set by the latest version of the *Declaration of Helsinki* were followed and conformed to.

### 2.2 Study design

All participants completed a preliminary visit (involving a maximal ramp test) and a familiarization session, followed by a combined cooling intervention trial (COOL) and a control trial (CON) in a randomized, order‐balanced, within‐subject design. All three trials (familiarization, COOL and CON) were separated by 7 days and performed in a hot environment (33°C, 50% relative humidity) in line with temperatures from previous events, such as 2021 Tokyo Olympics. Additionally, COOL and CON were completed at the same time of the day to eliminate the effect of circadian rhythm (Van Dongen & Dinges, 2005). Participants were required to arrive to the laboratory 2 h postprandial, consume a minimum of 500 mL of water, avoid caffeine prior to arrival and avoid strenuous exercise and alcohol for 24 h prior to the main trial. Diet was replicated in the 24 h prior to the experimental trials.

### 2.3 Maximal ramp test

To calculate the maximal power and maximal oxygen uptake of each participant, a maximal ramp test was used in a preliminary visit, which was conducted in moderate conditions. Participants began cycling at an intensity of 100 W, which increased by 15 Watts every 1 min. In the final 15 s of each 1 min stage, the heart rate and rate of perceived exertion (RPE) were collected. When the participant was approaching volitional exhaustion and could continue for only 1 min more, an expired air sample was taken via a Douglas bag. After the cessation of exercise, the expired air sample was analysed to calculate the maximal oxygen uptake, and a capillary sample was taken 5 min after the end of the test for the determination of blood lactate concentration. To ensure that a true maximum was achieved, two of the three following criteria were met; a postexercise blood lactate concentration of ≥8 mmol L^−1^, reached age‐predicted maximum heart rate [calculated as 220 minus age (in years)], and an RPE of ≥18.

### 2.4 Familiarization

The familiarization replicated the COOL protocol; however, the participants completed only the first half of the high‐intensity intermittent cycling protocol. In particular, to familiarize participants with the sprint protocol, the first two sprints were completed at 50% of the participant's maximum effort, the third and fourth sprint were completed at 75% of the participant's maximum effort, with sprints 6–10 at full effort.

### 2.5 Main trials

**FIGURE 1:**
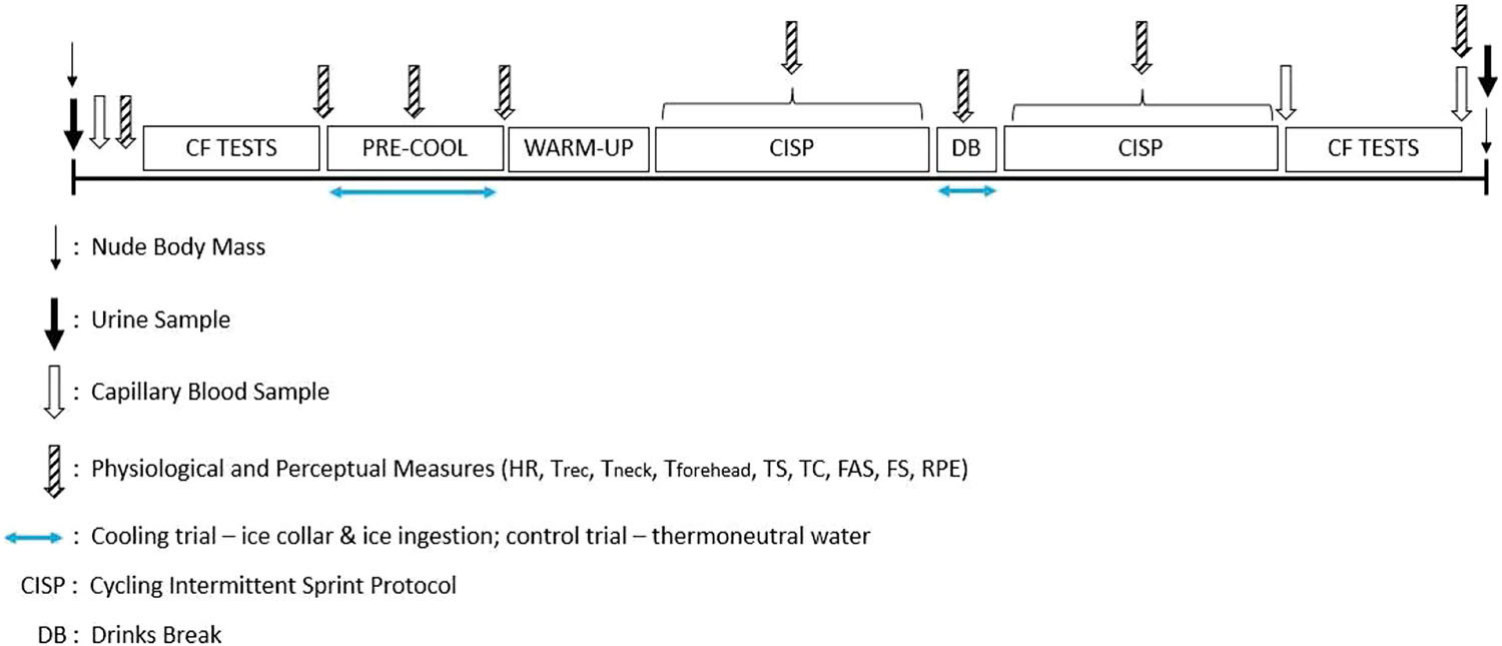
Schematic diagram showing the protocol of the main trials. Abbreviations: CF tests, cognitive function tests; CISP, cycling intermittent sprint protocol; DB, drinks break; FAS, felt arousal scale; FS, feeling scale; HR, heart rate; RPE, rating of perceived exertion; TC, thermal comfort; T_forehead_, forehead temperature; T_neck_, neck temperature; T_rec_, rectal temperature; TS, thermal sensation.

When participants arrived at the laboratory, nude body mass was recorded (WLK 150 Warrior Washdown Scales, Adam Equipment, Milton Keynes, UK), a urine sample was collected and analysed for urine osmolality, and a rectal probe was self‐inserted to measure core temperature. Following this, outside the environmental chamber, baseline physiological and perceptual measures were taken, and a capillary blood sample collected to measure baseline blood glucose and lactate concentrations. Participants entered the environmental chamber before completing a battery of cognitive function tests. Participants then sat, rested, in the environmental chamber for a pre‐cool period of 30 min with either the combined cooling intervention in COOL or with only thermoneutral water in CON. The participants were then required to complete a 4 min cycling warm‐up at their target recovery power before starting the high‐intensity intermittent cycling protocol. After the cessation of the cycling, a capillary blood sample was taken before and after the final cognitive function tests. A urine sample and nude body mass were recorded once participants had exited the environmental chamber. Throughout the main trials, physiological and perceptual measures were collected at various time points (Figure 1).

#### 2.5.1 High‐intensity intermittent cycling protocol

The high‐intensity intermittent cycling protocol used in the present study was modified from previously published work (Castle et al., 2006; Hayes et al., 2013) and aimed to replicate the demands of one‐half of a competitive team sport match. It consisted of two sets of ten 2 min stages of cycling, separated by a 4 min drinks break. Each 2 min stage involved a 5 s maximal sprint against a resistance previously determined based on Wattbike recommendations (Wattbike, Nottingham, UK), followed by 1 min 45 s of active recovery and 10 s of rest. During the active recovery phase of each stage, participants were required to cycle at 35% of their maximal power that was previously calculated from the maximal ramp test. The variables of interest for the high‐intensity intermittent cycling were peak and mean power output, in addition to recovery power output, which were recorded for each sprint phase.

#### 2.5.2 Combined cooling method

The present study involved a combined cooling strategy, which consisted of ice slurry ingestion and an ice collar. The ice slurry consisted of 50% water and 50% ice, weighing a total of 7.5 g kg^−1^ of body mass during the pre‐cool and 1 g kg^−1^ body mass during the drinks break. During the pre‐cool, the total weight of the ice slurry was divided into three separate drinks, given to participants in 10 min intervals. The ice collar consisted of three cups of ice (230 g per cup) placed into a cotton sheet and secured with cable ties, which was worn around the neck during the pre‐cool, then re‐made and worn during the drinks break. During CON, participants received the same weight in thermoneutral water (∼37°C) and did not receive the ice collar during the pre‐cool and drinks break.

### 2.6 Measurements

#### 2.6.1 Cognitive function tests

All cognitive function tests in the study were completed in the same order, carried out on a laptop computer (Lenovo ThinkPad, Lenovo PC HK Ltd, China), using a custom‐made software package, and took place before the pre‐cool period and after the high‐intensity intermittent cycling. The participants wore ear defenders to minimize external distractions whilst completing the tests. Before the tests, the participants were provided with three to six practice stimuli to allow for re‐familiarization and negate any potential learning effects, which is in line with previous research (Malcolm et al., 2018). Although there was feedback provided for the practice stimuli, these data were discarded, and no feedback was given once the test started. For all cognitive tests, the variables of interest were the response accuracy, which was the percentage of correct responses, and response time of correct responses, which was the time between presentation of stimuli and the response. Minimum and maximum cut‐offs for response time were used for each test and were based on excluding unreasonably fast (anticipatory) responses (<100 ms) and undue slow responses (1500–4000 ms, depending on task complexity), as used in previous research (Cowe et al., 2024). This battery of cognitive tests was chosen because it reflects the domains that are relevant to team sports, which are crucial for performance and success in competition and aid athletes in managing varying environments and situations (Williams et al., 2011; Yongtawee et al., 2021).

#### 2.6.2 Visual search

To measure perception and visual processing, the visual search test was used, as in previous research (e.g., Malcolm et al., 2018). It included a simple level and a complex level, each consisting of 21 stimuli. The simple level involved participants responding as quickly as possible when a green, bold, outlined triangle appeared on a black screen by pressing the space bar. During the complex level, participants were required to respond when a triangle shape made of several dots appeared on the screen. To induce a flickering effect, the screen was covered in green dots, which created the background and were redrawn every 250 ms.

#### 2.6.3 Stroop task

The executive function of the participant and their ability to supress automated responses (inhibitory control) were measured with the Stroop task (Stroop, 1935), as used in previous research (e.g., Cowe et al., 2024), which involved a congruent level and an incongruent level, consisting of 20 and 40 stimuli, respectively. The congruent level presented a word written in white ink in the centre of a black screen, and the participants were required to choose the word on the left or right of the screen, using the arrow keys, that matched the central word. During the incongruent level, participants were required to choose the word on the left or right of the screen that matched the ink colour of the central word, instead of the word itself.

#### 2.6.4 Sternberg paradigm

To measure working memory of the participant, the Sternberg paradigm (Sternberg, 1966) was used, which involved three levels and differing amounts of items to remember: one item, three items or five items (e.g., Cowe et al., 2024). The one‐item level involved 16 stimuli, whereas the three‐item and five‐item levels each involved 32 stimuli. The participants were required to choose whether the number/letter presented on the screen was a target or a distractor from the initial numbers/letters they were shown at the beginning of each level. If the number/letter presented was a target, they pressed the right arrow, whereas if it was a distractor, they pressed the left arrow. On the one‐item level, the target was always ‘3’, whereas on the three‐ and five‐item levels, the targets were always letters and were generated randomly.

#### 2.6.5 Rapid visual information processing

The rapid visual information processing (RVIP) test measured the sustained attention of the participant (Hilti et al., 2010) and lasted for 5 min. The test involved numbers from 2 to 9 appearing on the screen at 800 ms intervals, with eight target sequences per minute, equalling 40 target stimuli. When a sequence of three consecutive odd or even numbers appeared on the screen (e.g., 4–2–6; 7–3–5), participants were instructed to press the space bar. Correct responses were registered only whilst the final digit of a target sequence was presented and in the subsequent 1500 ms.

#### 2.6.6 Physiological measures

Rectal temperature was measured using a self‐inserted rectal probe (MEAS 4400 series temperature probe, Measurement Specialities Inc., MN, USA), which was inserted 10 cm past the anal sphincter. A core temperature logger was used to record core temperature (4600 thermometer, Measurement Specialities Inc.). Neck and forehead temperature were measured from the midpoint of the neck and forehead (RS 51 digital thermometer, RS Pro, RS Components Ltd, Corby, UK). Heart rate was recorded using a watch and chest‐worn heart rate strap (Sigma Sports Ltd, Kingston Upon Thames, UK). Blood glucose and lactate concentrations were collected via a capillary blood sample and analysed using an automated blood analyser (Biosen C, EKF Diagnostics, Barleben, Germany). To measure urine osmolality, pre‐ and post‐trial urine samples were collected and analysed using a pocket refractometer (Atago Co. Ltd, Japan). Whole‐body sweat rate was calculated by dividing sweat loss [(pre body mass − post body mass) + fluid intake − urine output] by the duration of the trial (120 min; Baker et al., 2018).

#### 2.6.7 Perceptual measures

Ratings of perceived exertion were measured on a scale from 6 (very, very light) to 20 (very, very hard) (Morgan & Borg, 1976). Participants provided ratings of thermal sensation on a scale from 0 (unbearably cold) to 8 (unbearably hot) (Casa et al., 2007). Thermal comfort was measured on a scale from −3 (much too cold) to +3 (much too warm) (Bedford, 1936), and feelings of arousal were measured using a scale from 1 (low activation) to 6 (high activation) (Svebak & Murgatoyd, 1985). Finally, ratings of feeling were measured using a scale of −5 (very bad) to +5 (very good) (Hardy & Rejeski, 1989). All perceptual scales have been proved to be valid (Bedford, 1950; Brito et al., 2022; Hardy & Rejeski, 1989; Pereira et al., 2011; Toner et al., 1986) and used in previous studies of this nature (e.g., Cowe et al., 2024; Malcolm et al., 2018).

### 2.7 Statistical analyses

All intermittent cycling performance, physiological and perceptual data in the present study were analysed using the Statistical Packages for Social Sciences (SPSS, v.29.0, Chicago, IL, USA) through a two‐way repeated‐measures ANOVA (trial × time). *Post hoc* analyses with Bonferroni corrections were conducted for all significant trial × time interactions, and the delta values are reported as the mean ± SD. Normality was checked via visual inspection using histograms and Q–Q plots. The cognitive function data were analysed using the open‐source software R (www.R‐project.org), using mixed effect models. Similar to previous research (e.g., Cooper et al., 2015; Cowe et al., 2024), separate analyses were performed for each test and level, owing to the varying levels of cognitive processing required. All analyses were performed using a two‐way repeated‐measures (trial × time) approach. For response time, analyses were conducted using the *lme* package (yielding *t* statistics), whereas for accuracy, analyses were conducted using the *glmer* package (yielding *z* statistics), owing to the binomial nature of the accuracy data. For the cognitive data, the raw effect sizes have been calculated (response time in milliseconds and accuracy as the percentage correct), both as the mean difference and the standard error of this difference. Effect sizes (Cohen's *d*) were calculated for the all the intermittent cycling performance, physiological and perceptual variables using trial pairings and interpreted according to convention (<0.2 = trivial effect; 0.2 to <0.5 = small effect; 0.5 to <0.8 = medium effect; and ≥0.8 = large effect; Cohen, 1992). For all statistical analyses, significance was accepted at the level of *p* < 0.05, and all data are presented as the mean ± SD.

## 3 RESULTS

### 3.1 Power output

No differences were seen in peak, mean or recovery power output between CON and COOL (main effects of trial, peak: *p* = 0.153, *d* = 0.08; mean: *p* = 0.176, *d* = 0.07; recovery: *p* = 0.683, *d* = 0.02); however, peak and mean power output increased over time in both trials, whereas recovery power output decreased (main effect of time, peak: *F*_19,532_ = 4.345, *p *< 0.001; mean: *F*_19,532_ = 2.996, *P *< 0.001; recovery: *F*_19,513_ = 12.331, *p *< 0.001). Moreover, the pattern of change in peak, mean or recovery power output also did not differ between the two conditions (trial × time interactions, peak: *p* = 0.230; mean: *p* = 0.426; recovery: *p* = 0.758; Appendix Tables A1 and A2).

### 3.2 Perceptual measures

#### 3.2.1 Thermal sensation

**FIGURE 2:**
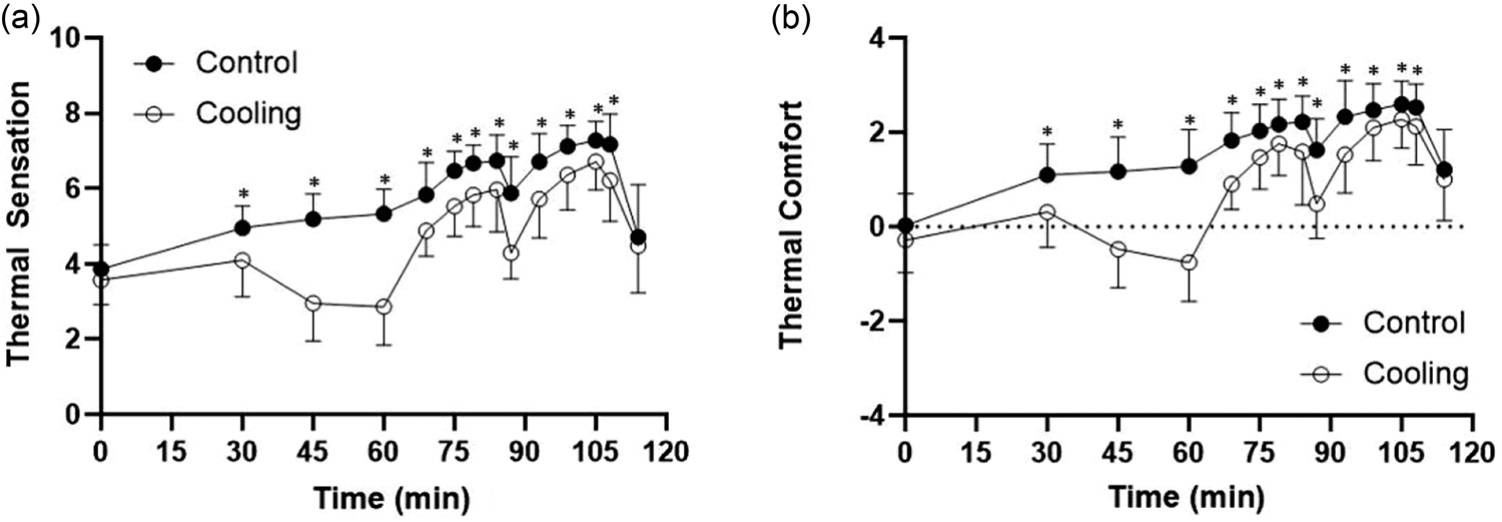
Ratings of thermal sensation (a) and thermal comfort (b) during CON and COOL (mean ± SD) from 29 participants. (a) Thermal sensation main effect of trial: *p* < 0.001, *d* = 0.70; main effect of time: *p* < 0.001; trial × time interaction: *p* < 0.001. (b) Thermal comfort main effect of trial: *p* < 0.001, *d* = 0.70; main effect of time: *p* < 0.001 trial × time interaction: *p* < 0.001. *Significant difference.

**FIGURE A1:**
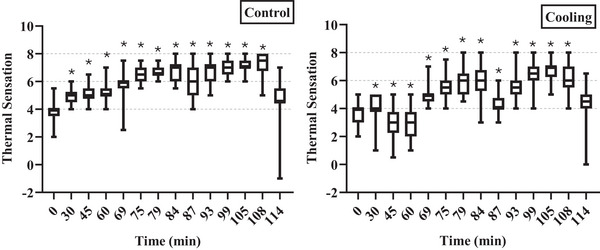
Box plots of the ratings of thermal sensation during CON and COOL from 29 participants. Thermal sensation main effect of trial: *p* < 0.001, *d* = 0.7; main effect of time: *p* < 0.001; trial × time interaction: *P* < 0.001; *Significant difference.

Participants reported lower levels of thermal sensation in COOL (5 ± 2) in comparison to CON (6 ± 1) (main effect of trial, *F*_1,28_ = 112.992, *p* < 0.001, *d* = 0.70), and this increased over time (main effect of time, *F*_13,364_ = 175.284, *p* < 0.001). Furthermore, a trial × time interaction was seen, whereby thermal sensation was lower from the start of the pre‐cool until the cessation of exercise in COOL compared with CON (trial × time interaction, *F*_13,364_ = 13.070, *p* < 0.001; COOL change: 2.64 ± 1.28, CON change: 3.31 ± 1.02; Figure 2; Appendix Figure A1).

#### 3.2.2 Thermal comfort

**FIGURE A2:**
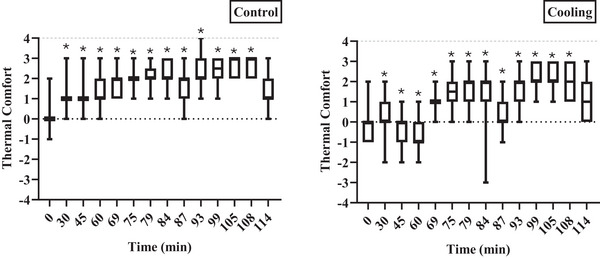
Box plots of the ratings of thermal comfort during CON and COOL from 29 participants. Thermal comfort main effect of trial: *p* < 0.001, *d* = 0.7; main effect of time: *p* < 0.001; trial × time interaction: *p* < 0.001. *Significant difference.

In COOL, lower ratings of thermal comfort were recorded (1 ± 1) compared with CON (2 ± 1; main effect of trial, *F*_1,28_ = 92.428, *p* < 0.001, *d* = 0.70), and these ratings increased over time (main effect of time, *F*_13,364_ = 96.617, *p* < 0.001). Moreover, thermal comfort was lower from the beginning of the pre‐cool until the cessation of exercise in COOL (trial × time interaction, *F*_13,364_ = 13.136, *p* < 0.001; COOL change: 2.40 ± 1.18, CON change: 2.50 ± 0.89; Figure 2; Appendix Figure A2).

#### 3.2.3 Rating of perceived exertion

**FIGURE 3:**
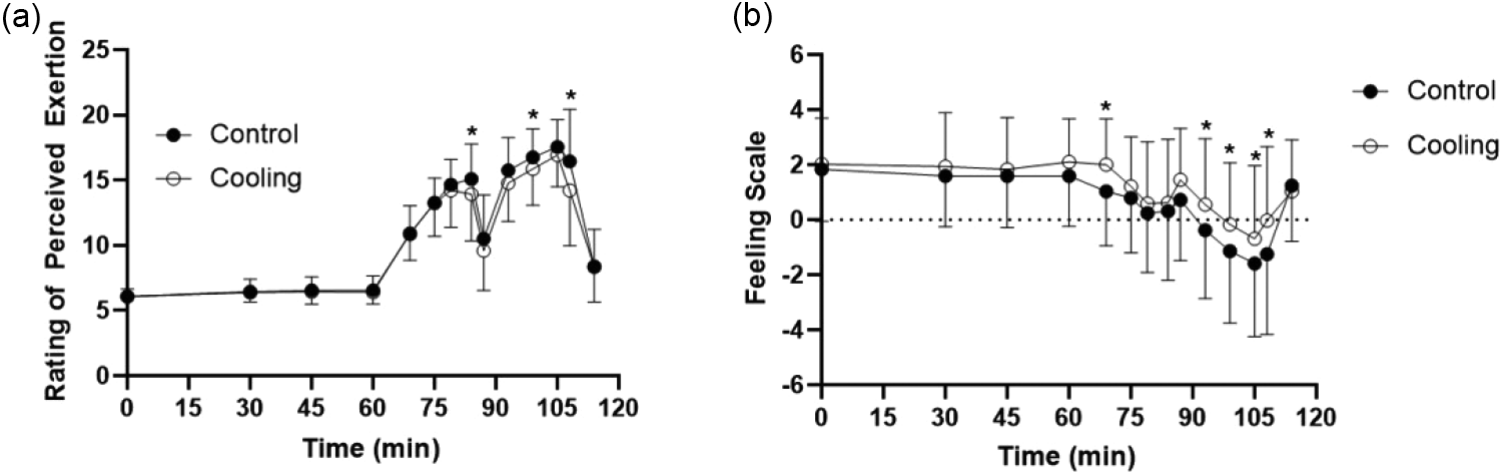
Ratings of perceived exertion (a) and feeling (b) during CON and COOL (mean ± SD) from 29 participants. (a) Rating of perceived exertion main effect of trial: *p* = 0.064, *d* = 0.12; main effect of time: *p* < 0.001, trial × time interaction: *p* < 0.001. (b) Feeling scale main effect of trial: *p* = 0.017, *d* = 0.40; main effect of time: *p* < 0.001, trial × time interaction: *p* = 0.016. *Significant difference.

**FIGURE A3:**
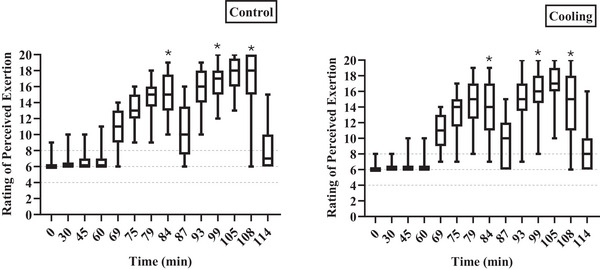
Box plots of ratings of perceived exertion during CON and COOL from 29 participants. Rating of perceived exertion main effect of trial: *p* = 0.064; main effect of time: *p* < 0.001; trial × time interaction: *p* < 0.001. *Significant difference.

Ratings of perceived exertion did not differ between COOL and CON (main effect of trial, *p* = 0.064, *d *= 0.12); however, they did increase over time (main effect of trial, *F*_13,364_ = 175.284, *p* < 0.001). There was a significant difference in the pattern of change across the trials (*F*_13,364_ = 3.684, *p* < 0.001; COOL change: 8.14 ± 4.33, CON change: 10.34 ± 4.04; Figure 3). Ratings of perceived exertion were lower in COOL at the beginning of the drinks break, halfway through the second set of cycling stages and at the cessation of exercise, when compared with CON (Figure 3a; Appendix Figure A3).

#### 3.2.4 Feeling scale

**FIGURE A4:**
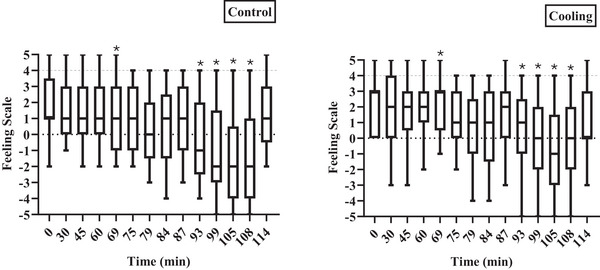
Box plots of ratings of feeling during CON and COOL from 29 participants. Feeling scale main effect of trial: *p* = 0.017, *d* = 0.4; main effect of time: *p* < 0.001; trial × time interaction: *p* = 0.016. *Significant difference.

Ratings by participants on the feeling scale were higher in COOL (1 ± 2) compared with CON (0 ± 3; main effect of trial, *F*_1,28_ = 6.488, *p* = 0.017, *d* = 0.40) and decreased over time (main effect of time, *F*_13,364_ = 27.704, *p* < 0.001). Ratings on the feeling scale were higher in COOL compared with CON at the start of exercise and for the entirety of the second set of cycling (trial × time interaction, *F*_13,364_ = 2.056, *p* = 0.016; COOL change: −2.07 ± 2.59, CON change: −3.07 ± 2.69; Figure 3b; Appendix Figure A4).

#### 3.2.5 Felt arousal scale

Between the two trials, there were no overall differences in ratings of arousal (main effect of trial, *p* = 0.152, *d *= 0.13); however, they did decrease over time (main effect of time, *F*_13,564_ = 3.967, *p* < 0.001). Moreover, the pattern of change in arousal did not differ over time (trial × time interaction, *p* = 0.184).

### 3.3 Cognitive function

The cognitive function data (response time and accuracy) for each test, separated by trial, time point and test level, are displayed in Table 1. The effect sizes for the main effect of trial, time and trial × time interaction for each test, test level and variable of interest can be found in the Appendix (Table A3).

**TABLE 1:** Response times (in milliseconds) and accuracy (as a percentage) for the battery of cognitive tests completed pre‐ and postexercise by 29 participants. All data reported as mean ± SD.

			Control trial		Cooling trial		*P*‐value		
Test	Variable	Test level	Pre‐exercise	Postexercise	Pre‐exercise	Postexercise	Trial effect	Time effect	Interaction effect
Visual search	Response time (ms)	Simple	540 ± 52	536 ± 43	538 ± 45	544 ± 44	0.916	0.223	0.187
		Complex	1408 ± 275	1289 ± 244	1373 ± 270	1358 ± 269	0.194	0.674	0.009*
	Accuracy (%)	Simple	98.9 ± 2.0	99.2 ± 1.8	99.4 ± 1.6	98.9 ± 2.0	0.374	0.374	0.298
		Complex	99.2 ± 2.1	99.2 ± 2.1	99.7 ± 1.2	99.0 ± 1.9	0.275	0.179	0.290
Stroop task	Response time (ms)	Simple	628 ± 102	621 ± 127	624 ± 88	620 ± 125	0.167	0.744	0.429
		Complex	818 ± 183	807 ± 201	820 ± 187	772 ± 164	0.473	0.001*	0.002*
	Accuracy (%)	Simple	98.2 ± 2.8	97.5 ± 4.6	97.9 ± 3.2	97.5 ± 3.5	0.753	0.457	0.958
		Complex	96.2 ± 3.6	96.1 ± 2.8	96.3 ± 2.8	95.5 ± 4.6	0.718	0.613	0.911
Sternberg paradigm	Response time (ms)	One‐item	450 ± 83	424 ± 85	437 ± 75	431 ± 78	0.046*	0.344	0.015*
		Three‐item	524 ± 79	501 ± 79	529 ± 80	521 ± 91	0.431	0.086	0.076
		Five‐item	605 ± 81	580 ± 90	621 ± 119	606 ± 119	0.257	0.091	0.207
	Accuracy (%)	One‐item	97.8 ± 3.5	97.8 ± 3.9	97.5 ± 3.5	98.0 ± 3.4	*P* = 0.824	*P* = 0.649	0.747
		Three‐item	97.8 ± 2.5	96.1 ± 3.3	96.8 ± 2.6	96.7 ± 3.5	*P* = 0.192	*P* = 0.894	0.160
		Five‐item	96.8 ± 3.6	95.6 ± 5.0	96.3 ± 3.2	95.2 ± 4.6	*P* = 0.602	*P* = 0.238	0.927
RVIP	Response time (ms)		490 ± 84	463 ± 65	491 ± 68	484 ± 91	*P* = 0.640	*P* = 0.062	0.431
	Accuracy (%)		55.9 ± 20.9	58.7 ± 22.4	56.8 ± 19.8	60.7 ± 23.2	0.408	0.072	0.897

*Note*: All data are reported as the mean ± SD.

#### 3.3.1 Visual search

On the simple level of the visual search test, no differences were seen in response times between CON and COOL (main effect of trial, *p* = 0.916), nor did it change over time (main effect of time, *p* = 0.223). The pattern of change also did not differ (trial × time interaction, *p* = 0.187). Although there was no difference in response times on the complex level of the visual search test between the trials (main effect of trial, *p* = 0.194) or over time (main effect of time, *p* = 0.674), response times became quicker over time to a greater extent in CON compared with COOL (trial × time interaction, *t*_2299_ = −2.629, *p* = 0.009; COOL change: −14.86 ± 233.96 ms, CON change: −118.14 ± 192.53 ms; Table 1).

Accuracy did not differ between the trials or over time for either the simple or complex level of the visual search test (main effect of trial, simple: *p* = 0.374; complex: *p* = 0.275; main effect of time, simple: *p* = 0.374; complex *p* = 0.179). Moreover, the pattern of change across both levels was also not different between CON and COOL (trial × time interactions, simple: *p* = 0.298; complex: *p* = 0.290).

#### 3.3.2 Stroop task

No differences were seen in response times on the simple level of the Stroop task (main effect of trial, *p* = 0.167; main effect of time, *p *= 0.744), and the pattern of change was not different between CON and COOL (trial × time interaction, *p *= 0.429). Despite response times not being different between the trials on the complex level of the Stroop task (main effect of trial, *p *= 0.473), response times were quicker over time (main effect of time, *t*_4634_ = −5.999, *p *< 0.001). Moreover, response times became quicker to a greater extent over time in COOL compared with CON (trial × time interaction, *t*_4634_ = 3.083, *p* = 0.002; COOL change: −48.54 ± 80.19 ms, CON change: −10.82 ± 96.38 ms; Table 1).

On both the simple and complex levels of the Stroop task, accuracy did not differ between the trials (main effects of trial, simple: *p* = 0.753; complex: *p* = 0.718; main effect of time, simple: *p *= 0.457; complex: *p *= 0.613), nor was the pattern of change different (trial × time interactions, simple: *p* = 0.958; complex: *p* = 0.911).

#### 3.3.3 Sternberg paradigm

Response times were found to be quicker in COOL (434 ± 77 ms) compared with CON (437 ± 84 ms) on the number level of the Sternberg paradigm (main effect of trial, *t*_1721_ = 1.998, *p* = 0.046; Table 1) but not over time (main effect of time, *p *= 0.344). Furthermore, the pattern of change between trials differed in that response times became quicker over time to a greater extent in CON compared with COOL (trial × time interaction, *t*_1721_ = −2.447, *p* = 0.015; COOL change: −5.57 ± 61.42 ms, CON change: −26.79 ± 53.60 ms). However, no differences were found between trials or over time in response times on the Sternberg paradigm three‐item and five‐item levels (main effects of trial, three‐item: *p* = 0.431; five‐item: *p* = 0.257; main effect of time, three‐item: *p *= 0.086; five‐item: *p *= 0.091), and the pattern of change over time was also similar between CON and COOL (trial × time interactions, three‐item: *p* = 0.076; five‐item: *p *= 0.207).

On the number, three‐item and five‐item levels of the Sternberg paradigm, there were no differences in accuracy between CON and COOL or over time (main effects of trial, number: *p* = 0.824; three‐item: *p* = 0.192; five‐item: *p* = 0.602; main effect of time, number: *p *= 0.649; three‐item: *p *= 0.894; five‐item: *p *= 0.238), and the pattern of change did not differ between the trials (trial × time interactions, number: *p *= 0.747; three‐item: *p* = 0.160; five‐item: *p* = 0.927).

#### 3.3.4 RVIP

For RVIP, no differences were found in response time between the trials or over time (main effect of trial, *p* = 0.640; main effect of time, *p *= 0.062), nor did the pattern of change differ between CON and COOL (trial × time interaction, *p* = 0.431). Likewise, accuracy was not different between the trials or over time (main effect of trial, *p* = 0.408; main effect of time, *p *= 0.072), and the pattern of change in accuracy also did not differ (trial × time interaction, *p* = 0.897).

### 3.4 Physiological measures

#### 3.4.1 Rectal temperature

**FIGURE 4:**
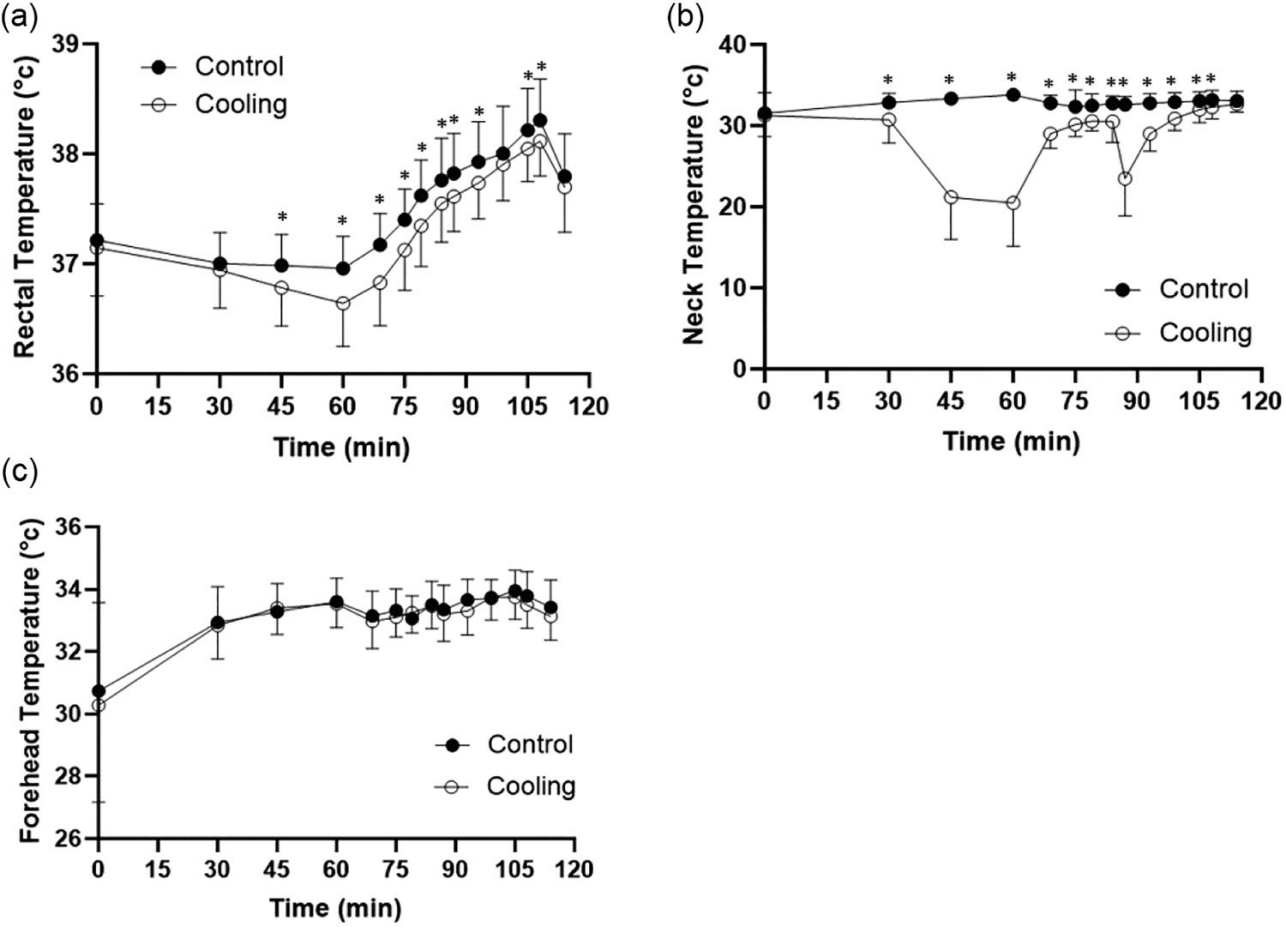
Rectal (a), neck (b) and forehead (c) temperature during COOL and CON (mean ± SD) from 29 participants. (a) Rectal temperature main effect of trial: *p* < 0.001, *d* = 0.3; main effect of time: *p* < 0.001, trial × time interaction: *p* < 0.001. (b) Neck temperature main effect of trial: *p* < 0.001, *d* = 1; main effect of time: *p* < 0.001, trial × time interaction: *p* < 0.001; (c) Forehead temperature main effect of trial: *p* = 0.134, *d* = 0.14; main effect of time: *p* < 0.001, trial × time interaction: *p* = 0.561. *Significant difference between trials.

**FIGURE A5:**
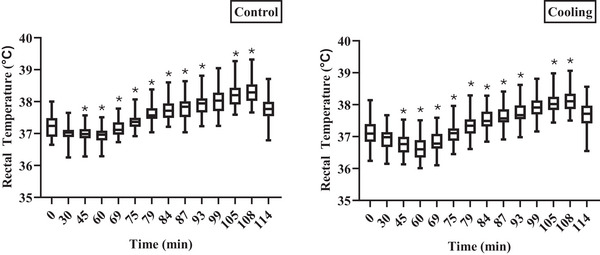
Box plots of rectal temperature during COOL and CON from 29 participants. Rectal temperature main effect of trial: *p* < 0.001, *d* = 0.3; main effect of time: *p* < 0.001; trial × time interaction: *p* < 0.001. *Significant difference between trials.

Lower rectal temperatures were recorded in COOL (average trial rectal temperature: 37.39°C ± 0.59°C) compared with CON (average trial rectal temperature: 37.59°C ± 0.56°C) (main effect of trial, *F*_1,27_ = 22.754, *p* < 0.001, *d* = 0.30) and did increase over time (main effect of time, *F*_13,351_ = 216.934, *p* < 0.001). Moreover, rectal temperature was significantly lower in COOL from halfway through the pre‐cool to the start of the second set of cycling and at the end of exercise (trial × time interaction, *F*_13,351_ = 3.965, *p* < 0.001; COOL change: 0.97°C ± 0.43°C; CON change: 1.30°C ± 0.34°C; Figure 4a; Appendix Figure A5). Additionally, a paired samples *t*‐test revealed that peak rectal temperature was lower in COOL (38.12°C ± 0.32°C) compared with CON (38.31°C ± 0.37°C) (*t*_27_ = 3.801, *p* < 0.001, *d* = 0.5).

#### 3.4.2 Neck temperature

**FIGURE A6:**
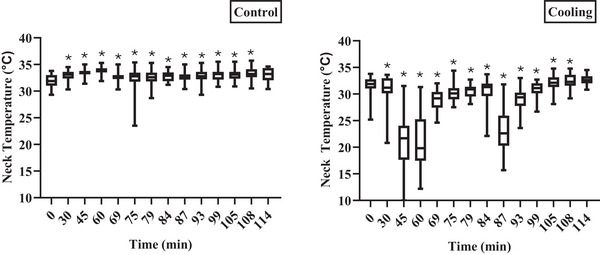
Box plots of neck temperature during COOL and CON from 29 participants. Neck temperature main effect of trial: *p* < 0.001, *d* = 1; main effect of time: *p* < 0.001; trial × time interaction: *p* < 0.001. *Significant difference between trials.

Neck temperature was found to be lower in COOL (average trial neck temperature: 28.87°C ± 4.87°C) when compared with CON (average trial neck temperature: 32.82°C ± 1.43°C) (main effect of trial, *F*_1,28_ = 141.739, *p* < 0.001, *d* = 1.00) and did increase over time (main effect of time, *F*_13,364_ = 56.904, *p* < 0.001). The pattern of change differed across the conditions (trial × time interaction, *F*_13,364_ = 74.770, *p* < 0.001; COOL change: 0.69°C ± 2.38°C; CON change: 1.31°C ± 1.79°C; Figure 4b; Appendix Figure A6). More specifically, after the start of the pre‐cool and at the cessation of exercise, lower neck temperatures were recorded in COOL (i.e., when the cooling collar was being worn).

#### 3.4.3 Forehead temperature

**FIGURE A7:**
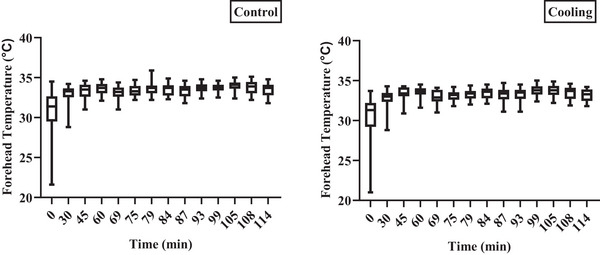
Box plots of forehead temperature during COOL and CON from 29 participants. Forehead temperature main effect of trial: *p* = 0.134; main effect of time: *p* < 0.001; trial × time interaction: *p* = 0.561.

Forehead temperature did not differ between COOL and CON (main effect of trial, *p* = 0.134, *d *= 0.14); however, it was seen to increase over time (main effect of time, *F*_13,364_ = 35.623, *p* < 0.001). Moreover, the pattern of change did not differ between the trials (trial × time interaction, *p* = 0.561; Figure 4c; Appendix Figure A7).

#### 3.4.4 Heart rate

**FIGURE 5:**
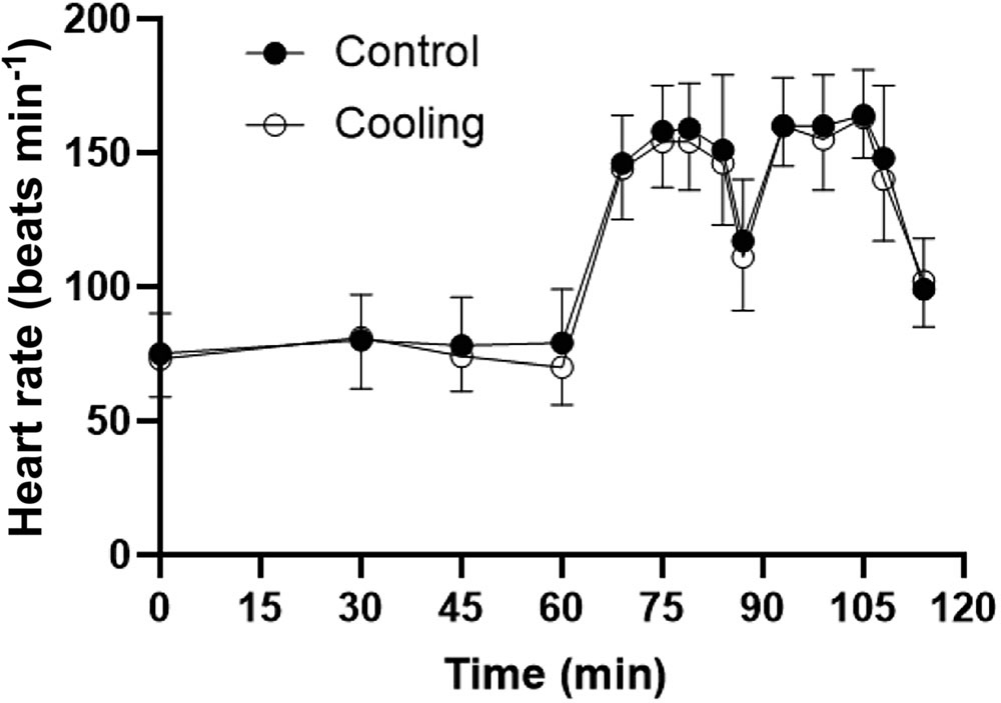
Heart rate during CON and COOL (mean ± SD) from 29 participants. Main effect of trial: *p* = 0.038, *d* = 0.10; main effect of time: *p* < 0.001; trial × time interaction: *p* = 0.092.

**FIGURE A8:**
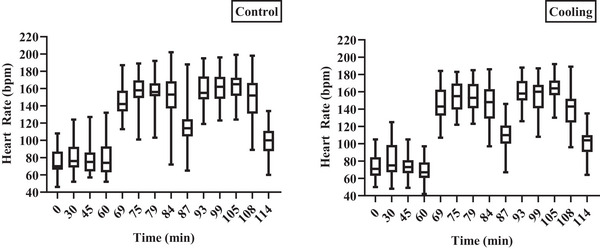
Box plots of heart rate during CON and COOL from 29 participants. Main effect of trial: *p* = 0.038, *d* = 0.1; main effect of time: *p* < 0.001; trial × time interaction: *p* = 0.092.

Although heart rate was overall lower in COOL (average trial heart rate: 123 ± 40 beats min^−1^) as opposed to CON (average trial heart rate: 127 ± 41 beats min^−1^; main effect of trial, *F*_1,28_ = 4.725, *p* = 0.038, *d* = 0.10; Figure 5; Appendix Figure A8) and did increase over time (main effect of time, *F*_13,364_ = 421.147, *p* < 0.001), the pattern of change in heart rate did not differ between the conditions (trial × time interaction, *p* = 0.092).

#### 3.4.5 Blood glucose and lactate

Blood glucose and lactate concentrations did not differ between the trials (main effects of trial, glucose: *p* = 0.442, *d *= 0.08; lactate: *p* = 0.483, *d *= 0.05), nor did the pattern of change differ (trial × time interactions, glucose: *p* = 0.813; lactate: *p* = 0.490). However, blood lactate concentrations were seen to increase over time (main effect of time, *F*_2,56_ = 109.448, *p* < 0.001).

#### 3.4.6 Whole‐body sweat rate

Whole‐body sweat rate was similar between the trials (average whole‐body sweat rate: control trial: 0.29 ± 0.12 L h^−1^; cooling trial 0.26 ± 0.13 L h^−1^ (*t*_28_ = −1.165, *p* = 0.254, *d *= 0.23).

## 4 DISCUSSION

The aim of the present study was to investigate the effect of internal and external cooling on high‐intensity intermittent cycling performance and cognitive function in the heat. The main findings were that repeated sprint performance was not affected by the combined cooling intervention. However, it was successful at lowering rectal and neck temperatures and heart rate. The participants also felt better and rated lower levels of thermal sensation, enhanced thermal comfort and lower perceived exertion in COOL. In terms of cognition, on the complex level of the Stroop task, response times became quicker to a greater degree during COOL. Response times were also quicker during COOL on the Sternberg paradigm number level. Yet, over time, response times became quicker to a greater extent in CON on the Sternberg number level, which was also the case for the complex level of the visual search test. Overall, these findings suggest that some domain‐specific effects of the heat and combined cooling intervention might be present.

### 4.1 Sprint performance

The combined cooling intervention did not elicit any differences in peak or mean power output during the high‐intensity intermittent cycling. This disagrees with previous research that found improvements in sprint performance following mixed‐method cooling (Minett et al., 2012). The underlying mechanisms involved in these previously found improvements relate to a reduction in core temperature, greater heat storage capacity and an increase in blood flow to the working muscles (Duffield & Marino, 2007; Girard et al., 2015). Despite a lower rectal temperature being found in COOL of the present study, these benefits are likely to manifest only when the exercise and thermal load are sufficient to induce heat strain, particularly increasing core temperatures to a critical level of 38.5°C or higher (Duffield & Marino, 2007; Sunderland & Nevill, 2005). Based on the peak rectal temperatures found in the present study, which were 38.12°C and 38.31°C in COOL and CON, respectively, it is possible that participants did not reach a level where sufficient heat strain was induced to impact power output and require the combined cooling (Parton et al., 2021). Moreover, high‐intensity exercise has been linked to increases in sweat rate owing to an elevated stimulation of the sweat glands (Mora‐Rodriquez et al., 2008). With higher sweat rates resulting in the development of hypohydration and this being associated with higher levels of physiological strain, it is understandable that individuals experience reductions in exercise performance when in a dehydrated state (Duffield et al., 2012; Maughan & Shirreffs, 2004; Maxwell et al., 2009; Montain & Coyle, 1992). However, this was not the case for the present study, because there were no differences observed in whole‐body sweat rate between the conditions. Therefore, this could potentially be another factor contributing to a lack of differences in peak and mean power output.

### 4.2 Perceptual responses

Lower ratings of thermal sensation and improved thermal comfort were reported during COOL, which is a common finding when investigating mixed‐method cooling (Mazalan et al., 2022b; Yanaoka et al., 2022). Coincidentally, thermal sensation and comfort were significantly lower in COOL at the same time points as neck temperature, which is the likely explanation for these improved perceptual responses (Cleary et al., 2014). The neck is an area of high allesthesial thermosensitivity, owing to a greater thermoreceptor density in this region; thus, cooling the neck is seen as an important intervention for making participants feel cooler (Cotter & Taylor, 2005).

Despite reduced thermal sensation tending to be coupled with improvements in exercise performance (Tyler et al., 2015; Yanaoka et al., 2022), this was not evidenced in the present study. It could be argued that although the differences in thermal comfort and sensation were statistically different, the one‐point difference on these perceptual scales might not have been large enough to elicit performance benefits in the intermittent cycling protocol (White et al., 2003). Additionally, previous research has found that exercise causes an increase in RPE regardless of cooling (Cleary et al., 2014), which is similar to the present study, in that there was no difference in RPE between the trials. However, an interaction was found, because RPE was statistically different at the end of the first set of cycling, halfway through the second set and at the cessation of exercise. Participants might have been aware that they were coming towards the end of a set of cycling, in addition to looking forwards to cooling at half‐time, felt motivated and, in turn, felt that the intermittent protocol was easier at that point. It is difficult to determine this, because motivation was not measured; however, this warrants further investigation.

### 4.3 Cognitive function

The findings of the present study suggest that the effects of the combined cooling intervention might be domain specific. For example, response times improved over time to a greater extent on the complex level of the Stroop task (a test of executive function) in COOL compared with CON. This finding is in line with previous research (Cowe et al., 2024; Wen et al., 2022) and might suggest that a player can choose the response more quickly when faced with opposing decisions in a real‐life match scenario (i.e., to dribble or pass; Malcolm et al., 2018). These improvements seen in response times might be linked to lower neck temperatures that have previously been associated with improvements in cognitive function performance when exercising in the heat, particularly in tasks of higher complexity (Lee et al., 2014).

On the contrary, in CON, response times became quicker to a greater extent on the Sternberg number level (a test of working memory) and on the complex level of the visual search test (a test of perception). These findings, coupled with the improvements in Stroop task response times in COOL mentioned above, indicate that the effects of heat and combined cooling differ for different domains of cognitive function. The potential reasoning for this is that higher‐order functions, such as executive function, might be more susceptible to heat stress owing to a limited amount of available attentional resources whilst exercising (Gaoua, 2010; Hocking et al., 2001; Schmit et al., 2017). This leads to an inability to complete the task at hand in addition to combatting heat stress and therefore explains why cooling might have influenced this domain of cognitive function (Gaoua et al., 2011; Schmit et al., 2017). The differences in response times seen in CON for the Sternberg paradigm and the visual search test might instead have been linked to slight increases in core temperature that have been found to improve cognitive performance through rises in arousal and cerebral blood flow (Grego et al., 2004; Schmit et al., 2017). More specifically, cerebral blood flow is responsible for delivering oxygen and nutrients to brain regions associated with cognitive function, such as the prefrontal cortex, to be able to maintain and potentially enhance cognitive performance (Komiyama et al., 2020; Ogoh et al., 2014).

### 4.4 Physiological responses

Despite limited effects on sprint and cognitive function performance, the combined cooling intervention was successful at improving some elements of physiological strain. This was evidenced through lower rectal and neck temperatures and heart rate, which are common findings when implementing combined cooling during intermittent exercise in the heat (Duffield et al., 2009; Fenemor et al., 2022). Lower rectal and neck temperatures are likely to be caused by the location of the cooling methods; in particular, ingesting cold drinks can reduce core temperature through the enthalpy of fusion (Siegel et al., 2012). This mechanism relates to altering the state of water from solid to liquid, which allows more heat to be transferred into the drink as opposed to being stored in the body. This is because the ice acts as an additional heat sink, thus reducing core temperature (Siegel et al., 2010). In addition, with the neck being an area of close proximity to the thermoregulatory centre in the brain and thermoreceptors being located in the gastrointestinal region, the cooling might have positively influenced the inhibitory feedback on core temperature (Haymaker, 1969; Morris et al., 2014; Villanova et al., 1997). These findings highlight the advantages of implementing a combined cooling intervention on the physiological strain that is likely to be experienced when performing intermittent exercise in the heat.

## 5 CONCLUSION

To conclude, the present study highlighted some potential domain‐specific effects of the combined cooling intervention through enhanced executive function, which is an important cognitive domain for successful sporting performance. Moreover, improved physiological and perceptual responses to intermittent exercise in the heat were observed, most probably owing to the location of the cooling methods. This is insightful for real‐world team sports, because these measures reflect the demand, both physiologically and perceptually, that athletes are exposed to when competing in the heat. However, the combined cooling intervention did not affect intermittent sprint performance, which was evidenced through a lack of differences in peak or mean power output.

## AUTHOR CONTRIBUTIONS

Stacey Cowe—conception and design of the work; acquisition, analysis and interpretation of the data; and writing the main body of work. Simon B. Cooper—conception and design of the work; analysis of data for the work; and drafting the work. Rachel Malcolm—conception and design of the work; and drafting the work. Caroline Sunderland—conception and design of the work; and drafting the work. All authors approved the final version of the manuscript and agree to be accountable for all aspects of the work in ensuring that questions related to the accuracy or integrity of any part of the work are appropriately investigated and resolved. All persons designated as authors qualify for authorship, and all those who qualify for authorship are listed.

## CONFLICT OF INTEREST

None declared.

## FUNDING INFORMATION

No funding was received for the present study.

6

**TABLE A1:** Peak, mean and recovery power output (in watts) during the first half (10 sprints) of the high‐intensity intermittent cycling protocol.

Power output (W)	SP1		SP2		SP3		SP4		SP5		SP6		SP7		SP8		SP9		SP10	
	CON	COOL	CON	COOL	CON	COOL	CON	COOL	CON	COOL	CON	COOL	CON	COOL	CON	COOL	CON	COOL	CON	COOL
Peak	1067 ± 219	1081 ± 173	1101 ± 201	1090 ± 176	1098 ± 202	1110 ± 176	1089 ± 209	1104 ± 181	1089 ± 194	1091 ± 197	1089 ± 190	1087 ± 197	1082 ± 192	1084 ± 200	1081 ± 195	1084 ± 208	1038 ± 170	1081 ± 199	1060 ± 211	1076 ± 207
Mean	904 ± 178	878 ± 213	923 ± 192	896 ± 223	916 ± 203	904 ± 230	912 ± 177	900 ± 219	927 ± 176	908 ± 225	931 ± 173	905 ± 231	934 ± 175	902 ± 230	919 ± 173	898 ± 236	887 ± 179	870 ± 230	909 ± 184	886 ± 238
Recovery	86 ± 18	89 ± 17	84 ± 20	88 ± 17	86 ± 20	85 ± 19	89 ± 18	89 ± 18	89 ± 19	88 ± 18	85 ± 20	84 ± 19	86 ± 18	87 ± 19	84 ± 19	83 ± 21	84 ± 20	87 ± 19	82 ± 18	83 ± 20

*Note*: All data are reported as the mean ± SD.

**TABLE A2:** Peak, mean and recovery power output (in watts) during the second half (10 sprints) of the high‐intensity intermittent cycling protocol.

Power output (W)	SP11		SP12		SP13		SP14		SP15		SP16		SP17		SP18		SP19		SP20	
	CON	COOL	CON	COOL	CON	COOL	CON	COOL	CON	COOL	CON	COOL	CON	COOL	CON	COOL	CON	COOL	CON	COOL
Peak	1049 ± 229	1078 ± 189	1088 ± 202	1111 ± 204	1045 ± 178	1088 ± 219	1057 ± 194	1062 ± 220	1040 ± 215	1056 ± 208	1044 ± 188	1037 ± 222	1042 ± 210	1067 ± 201	1019 ± 204	1039 ± 194	1025 ± 211	1085 ± 201	1095 ± 218	1104 ± 228
Mean	899 ± 208	893 ± 234	924 ± 176	907 ± 230	907 ± 167	890 ± 230	896 ± 175	869 ± 227	886 ± 187	871 ± 235	880 ± 186	857 ± 234	880 ± 217	872 ± 239	883 ± 188	851 ± 228	877 ± 196	883 ± 236	946 ± 200	918 ± 250
Recovery	85 ± 21	85 ± 19	84 ± 18	86 ± 18	79 ± 20	78 ± 22	78 ± 23	81 ± 23	79 ± 20	80 ± 25	74 ± 23	74 ± 25	80 ± 24	78 ± 22	75 ± 23	77 ± 24	74 ± 23	72 ± 26	73 ± 25	74 ± 24

*Note*: All data are reported as the mean ± SD.

**TABLE A3:** Raw effect sizes for the main effects of trial, time and trial × time interactions for all cognitive tests, separated by test level and variable of interest. Data presented as the mean difference (Mean Diff) and the standard error of this difference (Std Error).

Test	Variable	Test level	Trial		Time		Trial × time	
			Mean diff.	SE	Mean diff.	SE	Mean diff.	SE
Visual search	Response time (ms)	Simple	1.79	4.63	5.58	4.63	−9.09	6.56
		Complex	35.26	30.30	−18.49	30.28	−105.16	42.81
	Accuracy (%)	Simple	−0.56	0.63	0.56	0.63	0.90	0.86
		Complex	−0.92	0.84	−1.10	0.82	1.10	1.04
Stroop task	Response time (ms)	Simple	13.61	8.78	9.63	8.74	−11.62	12.44
		Complex	−6.50	10.81	−53.00	10.74	37.18	15.29
	Accuracy (%)	Simple	0.13	0.42	−0.28	0.38	0.03	0.56
		Complex	0.08	0.21	−0.10	0.20	0.03	0.29
Sternberg paradigm	Response time (ms)	One‐item	13.59	8.04	−6.20	8.03	−18.99	11.36
		Three‐item	−3.90	8.10	−6.81	8.13	−16.26	11.49
		Five‐item	−19.97	11.83	−9.05	11.88	−19.04	16.78
	Accuracy (%)	One‐item	0.10	0.45	0.21	0.46	−0.21	0.65
		Three‐item	0.38	0.29	−0.04	0.26	0.54	0.39
		Five‐item	0.13	0.26	−0.28	0.24	−0.03	0.34
RVIP	Response Time (ms)		−1.89	9.65	−19.01	9.43	−12.89	13.44
	Accuracy (%)		−0.07	0.09	0.16	0.09	−0.02	0.12

*Note*: Data presented as the mean difference (Mean diff.) and the standard error of this difference (SE). Interaction effect size represents the mean difference and standard error of the change over time on the hot trial compared with the change over time on the cool trial.

## Data Availability

The raw, anonymized data are available, upon request.
